# Comparative Effectiveness of Follitropin Delta, Follitropin Alpha, and hMG in ART Cycles: A Single‐Center Retrospective Cohort Study With Propensity Score Matching

**DOI:** 10.1002/rmb2.70060

**Published:** 2026-05-13

**Authors:** Noritoshi Enatsu, Kunihiro Enatsu, Yihsien Enatsu, Ai Yamada, Koji Yamada, Nao Hayashi, Eri Okamoto, Shoji Kokeguchi, Hiroaki Shibahara, Masahide Shiotani

**Affiliations:** ^1^ Hanabusa Women's Clinic Kobe city Japan

## Abstract

**Objective:**

To compare clinical outcomes of ART cycles stimulated with follitropin delta, follitropin alpha, or human menopausal gonadotropin (hMG) using a single‐center retrospective cohort with propensity score matching (PSM).

**Methods:**

We analyzed 20 809 ART cycles (2021–2024). PSM adjusted for age, body weight, anti‐Müllerian hormone, antral follicle count, prior ART attempts, stimulation protocol, and trigger method. The matched cohort comprised 7,647 cycles (2549/group). The primary outcome was oocyte yield. Secondary outcomes included blastocyst development, ovarian enlargement ≥ 5 cm, and clinical pregnancy rates.

**Results:**

The primary outcome regarding oocyte yield was statistically indeterminate across different analytical approaches, and the observed differences were clinically marginal. Both recombinant FSH preparations showed higher blastocyst yields and a lower observed incidence of ovarian enlargement ≥ 5 cm compared with hMG; the latter finding, however, does not establish a clinical safety advantage because clinically diagnosed OHSS was not evaluated. Clinical pregnancy rates were comparable among the three groups.

**Conclusions:**

Differences in oocyte yield among follitropin delta, follitropin alpha, and hMG were small and statistically inconsistent. Secondary findings should be interpreted as exploratory, suggesting broadly comparable clinical performance among the three gonadotropin preparations.

## Graphical abstract

In this large propensity score‐matched cohort study, the primary outcome regarding oocyte yield was statistically indeterminate across follitropin delta, alpha, and hMG. However, exploratory analyses suggest recombinant preparations were observationally associated with greater blastocyst development compared with hMG, though clinical safety benefits remain unestablished.

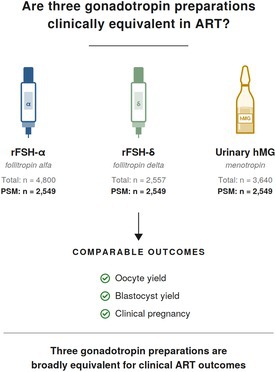

## 1 Introduction

Controlled ovarian stimulation (COS) constitutes an essential aspect of assisted reproductive technology (ART) cycles, as the appropriate stimulation strategies facilitate optimal follicular development and enable the retrieval of an adequate number of oocytes. Follicle‐stimulating hormone (FSH) preparations are commonly used in controlled ovarian stimulation (COS) for ovarian stimulation and have been incorporated into clinical practice following several advancements. Historically, during the 1950s, human menopausal gonadotropin (hMG) represented the primary pharmacological agent for ovulation induction. This compound, obtained from the urine of postmenopausal women, contains both FSH and luteinizing hormone (LH) in an approximate ratio of 1:0.33 [1]. Although hMG was effective in stimulating follicular development, its reliance on pooled human urine resulted in batch‐to‐batch variability and introduced potential risks associated with contaminant proteins and immunogenic impurities. In the 1960s, recognition that FSH is more critical to COS than LH drove further advancements in purification techniques, resulting in the production of urofollitropin, a urinary‐derived FSH preparation with significantly reduced LH contamination (FSH:LH ratio less than 1:0.0053) [2]. These refinements enabled more precise dosing and promoted consistent follicular growth during COS cycles. In the 1990s, the advent of recombinant DNA technology revolutionized gonadotropin therapy by enabling production of pure FSH in Chinese Hamster Ovary (CHO) cells. Products such as follitropin alfa and follitropin beta achieve 100% FSH purity and completely eliminate urinary proteins and LH [3]. These CHO‐derived recombinant FSH (r‐FSH) preparations offer superior lot‐to‐lot consistency, reduced immunogenic potential, and have demonstrated clinical efficacy and safety equivalent or superior to urinary‐derived formulations, with lower rates of ovarian hyperstimulation syndrome [4]. Most recently, follitropin delta has been produced in a human cell line (PER.C6). This human cell‐derived FSH features a unique glycosylation pattern rich in α2,6‐linked sialic acids compared with CHO‐derived r‐FSH [5]. This structural feature contributes to altered pharmacokinetics and underpins an individualized dosing algorithm based on patient body weight and serum anti‐Müllerian hormone (AMH) concentration [6]. Subsequent large‐scale phase III trials involving actual ART cycles have demonstrated that, across diverse populations, follitropin delta yields pregnancy and live birth rates comparable to those of Follitropin alpha or Follitropin beta, with a reduced risk of OHSS [7, 8].

Although these investigations were conducted as rigorous randomized controlled trials (RCTs) and provide considerable reliability, several limitations warrant consideration. Notably, the studies primarily examine fresh embryo transfer cycles. Additionally, participants assigned to the follitropin alpha or beta arms received a fixed initial dose of 150 IU, whereas those in the follitropin delta group began treatment with a dose determined by an algorithm. It should also be noted that all trials were sponsored by Ferring Pharmaceuticals, the manufacturer of follitropin delta. Therefore, large‐scale clinical evidence that is not subject to these limitations is required. This study aims to assess and compare clinical outcomes for patients treated with follitropin delta, follitropin alpha, and hMG within routine clinical settings, thereby offering insights into the efficacy of these products beyond the scope of controlled environments.

## 2 Materials and Methods

### 2.1 Patient Population

**FIGURE 1:**
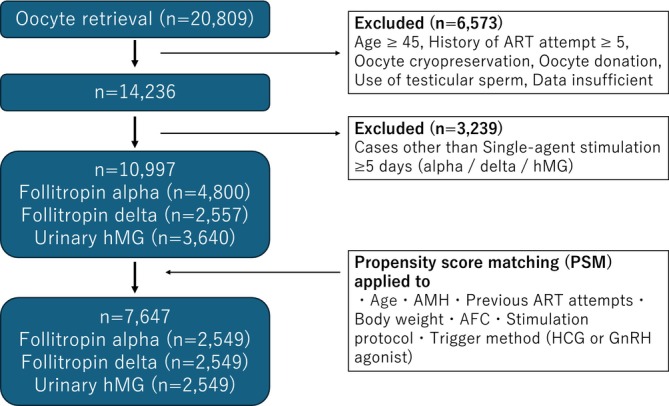
Flowchart of cohort selection, exclusion criteria, and the propensity score matching (PSM) process. The figure shows the initial number of oocyte retrieval cycles, the application of inclusion and exclusion criteria, allocation to stimulation agent groups (follitropin alpha, follitropin delta, and urinary hMG), and the final matched cohort. AMH indicates anti‐Müllerian hormone; AFC, antral follicle count; ART, assisted reproductive technology; hMG, human menopausal gonadotropin.

This retrospective study analyzed 20 809 ART cycles conducted at the institution between January 2021 and December 2024. As shown in Figure 1, cycles involving women aged 45 years or older, those with ≥ 5 previous ART attempts, oocyte donation and oocyte cryopreservation cycles were excluded. In addition, cycles that used testicular sperm were omitted to reduce potential male factor influences on IVF outcomes. Cycles missing key data, including serum AMH levels, FSH dosage, or duration of administration, were also excluded from the analysis. Consequently, 6573 cycles were excluded, leaving 14 236 cycles remaining.

During the subsequent phase, only stimulation cycles employing a single ovarian stimulation agent for a minimum duration of 5 days—namely follitropin alpha (Gonal‐f, Merck, Darmstadt, Germany), follitropin delta (Rekovelle, Ferring Pharmaceuticals, St‐Prex, Switzerland), or human menopausal gonadotropin hMG (Ferring, Ferring Pharmaceutical)—were chosen for inclusion in the analysis. This selection resulted in 4800 cycles allocated to the follitropin alpha group, 2557 cycles to the follitropin delta group, and 3640 cycles to the hMG group. In total, 10 997 cycles were incorporated into the next step.

### 2.2 Propensity Score Matching

To address the imbalance in sample size across treatment groups and reduce potential confounding, propensity score matching (PSM) was performed. Propensity scores were estimated using pairwise multivariable logistic regression models for each comparison (follitropin alpha vs. follitropin delta, follitropin alpha vs. hMG, and follitropin delta vs. hMG), with treatment assignment as the dependent variable. Covariates included age, body weight, anti‐Müllerian hormone (AMH), antral follicle count (AFC), history of ART attempts, ovarian stimulation protocol (PPOS, antagonist, or agonist), and trigger method (GnRH agonist or hCG/dual trigger). Age, AFC, and AMH are well‐established predictors of ovarian reserve and ART outcomes [9]. In addition, the number of prior ART attempts was incorporated because repeated IVF cycles are associated with a progressive decline in per‐cycle pregnancy rates [10]. Furthermore, the stimulation protocol and trigger method were included to adjust for physician selection bias regarding excessive ovarian response management. We then performed one‐to‐one nearest‐neighbor matching without replacement in two sequential steps, applying a caliper width of 0.2 of the standard deviation of the logit of the propensity score. First, the follitropin delta group was matched to the follitropin alpha group, and subsequently to the hMG group. Only those follitropin delta patients who were successfully paired in both comparisons, together with their corresponding follitropin alpha and hMG matches, were retained to constitute the final three‐group matched cohort (2549 cycles in each group). Covariate balance across the three groups was assessed using standardized mean differences (SMDs) for all covariates, with an absolute SMD < 0.10 considered indicative of adequate balance. The C‐statistic was also calculated to assess the model's discriminatory power and goodness of fit. Furthermore, the distributions of propensity scores before and after matching were visually inspected to ensure substantial overlap between the groups.

### 2.3 ART Cycle Management

A standard controlled ovarian hyperstimulation (COH) protocol was utilized for ovarian stimulation, consistent with established methodologies [11]. Oocyte stimulation involved the use of follitropin alpha, follitropin delta, or human menopausal gonadotropin (hMG). The cumulative gonadotropin dose was calculated by adding the amounts of FSH or hMG administered throughout the ovarian stimulation period. For patients receiving follitropin delta, dose units were expressed in micrograms (μg), while doses for other products were measured in international units (IU). To prevent premature LH surges, gonadotropin‐releasing hormone (GnRH) agonist, GnRH antagonist, or oral progesterone was used. Administration of the final oocyte maturation trigger, either GnRH agonist or recombinant human chorionic gonadotropin (r‐hCG), was performed when at least two follicles measured 17–18 mm in diameter or when the attending physician assessed the follicular cohort as mature. The dual trigger was applied when the initial oocyte retrieval yielded fewer mature oocytes than expected, indicating an inadequate response to the trigger.

### 2.4 Assessment of Ovarian Enlargement

Because clinically diagnosed ovarian hyperstimulation syndrome (OHSS) was not systematically evaluated in all cases, ovarian enlargement was assessed using a surrogate indicator defined as a maximum ovarian diameter ≥ 5 cm at the time of oocyte retrieval. This measure was selected to provide a uniform and objective assessment across all cycles. This surrogate does not represent clinically diagnosed OHSS and was not intended to capture the full clinical spectrum of OHSS.

### 2.5 Embryo Transfer Outcomes

Clinical pregnancies were evaluated in subsequent embryo transfer cycles. To reduce confounding variables, this study included only ET cycles involving single blastocyst transfers, while excluding those with early‐stage embryo transfers or multiple embryo transfers. In addition, given that our institution primarily employs frozen–thawed blastocyst transfer, fresh ET cycles were excluded. Frozen–thawed ET was performed either hormone replacement cycle or natural cycle protocol as we described previously [12]. Pregnancy was monitored using transvaginal ultrasound at 5 weeks gestational age, and detection of a fetal sac in the uterus was defined as clinical pregnancy. To ensure robust outcome assessment, the analysis included only those cases wherein more than 90 days had elapsed since the ET date. The ongoing pregnancy and live birth rates were determined according to patient status at the time of data collection.

### 2.6 Statistical Analysis

The primary outcome assessed in this study was the total number of oocytes retrieved, while secondary outcomes encompassed the incidence of ovarian enlargement ≥ 5 cm, the number of blastocysts generated and clinical pregnancy. Statistical comparisons of continuous variables between two groups were conducted using Welch's *t*‐test, whereas one‐way analysis of variance (ANOVA) was employed for comparisons involving three groups. Categorical variables were expressed as proportions and evaluated using the *χ* ^2^ test and risk ratio analysis, each accompanied by a 99% confidence interval. Statistical significance was defined as a *p*‐value < 0.01 or 99% confidence interval excluding 1.0.

For embryological outcomes, which involved multiple comparisons, the false discovery rate (FDR) was controlled using the Benjamini–Hochberg procedure, and both raw *p*‐values and FDR‐adjusted *q*‐values were reported. In addition, effect sizes were calculated using Cohen's *d* for continuous variables and risk ratios with 99% confidence intervals for categorical variables to aid interpretation of the clinical relevance of statistically significant findings.

To confirm the robustness of the findings, two sensitivity analyses were performed. First, cluster‐robust regression analysis was conducted in the matched cohort to account for the correlation of repeated cycles within the same patient (Table S1). Second, an inverse probability weighting (IPW) analysis was performed on the full unmatched cohort (*n* = 10 997) to assess consistency with the PSM results, specifically adjusting for body weight and all other covariates.

All statistical analyses were primarily performed using EZR (Saitama Medical Center, Jichi Medical University, Saitama, Japan), and RStudio (Posit, PBC, Boston, MA, USA), which are graphical user interfaces for R (The R Foundation for Statistical Computing, Vienna, Austria). Microsoft Excel (Microsoft 365, Microsoft Corporation, Redmond, WA, USA) was used only for basic descriptive calculations such as means and standard deviations.

### 2.7 Ethical Considerations

This study is a retrospective cohort analysis, and no interventions were made regarding the IVF treatment process. The Institutional Review Board of Hanabusa Women's Clinic, which includes members selected by the institution and an external third‐party institution, approved this study (approval number: 2025‐06). Patients provided informed consent prior to the treatment period preceding IVF cycles, and separate confirmation for data analysis and publication via anonymization was obtained independently of treatment consent. Only patients who consented to provide their data were included in the database.

## 3 Results

Table 1 summarizes the baseline patient characteristics after propensity score matching (PSM). A total of 7647 cycles were included, with 2549 cycles in each of the three groups (follitropin alpha, follitropin delta, and urinary hMG). No significant differences were observed among the three groups with respect to age, body weight, AMH, AFC, number of previous ART attempts, ovarian stimulation protocol, or trigger method. All Max SMD values were < 0.05, indicating excellent covariate balance. The C‐statistics for the propensity score models ranged from 0.62 to 0.73, indicating good discriminatory power and sufficient common support for matching. The propensity score distributions before and after matching are illustrated in Figure S1, demonstrating substantial overlap and sufficient common support across the groups.

**TABLE 1:** Baseline characteristics of patients before and after propensity score matching (PSM).

Variable	Before matching (Alpha/Delta/hMG)	Max SMD (before PSM)	After matching (Alpha/Delta/hMG)	Max SMD (after PSM)
Age (years)	36.5 (4.4)/35.7 (4.3)/37.3 (4.3)	0.37	35.6 (4.4)/35.7 (4.3)/35.6 (4.4)	0.04
Body weight (kg)	53.7 (9.0)/53.3 (8.2)/53.8 (8.3)	0.05	52.9 (8.4)/53.3 (8.2)/53.2 (7.6)	0.04
AMH (ng/mL)	2.5 (2.4)/2.6 (2.3)/2.3 (2.3)	0.11	2.6 (2.3)/2.6 (2.3)/2.6 (2.2)	0.03
AFC (n)	8.7 (6.0)/9.0 (5.9)/7.8 (5.5)	0.21	9.2 (5.8)/9.0 (5.8)/9.0 (5.2)	0.04
History of ART attempts (*n*)	2.1 (1.2)/1.9 (1.2)/2.2 (1.3)	0.20	2.0 (1.2)/2.0 (1.2)/2.0 (1.2)	0.02
Protocol: PPOS	36.1%/51.9%/34.9%	0.45	59.4%/60.0%/59.6%	0.02
Protocol: Antagonist	48.9%/42.0%/41.5%	0.21	36.6%/36.2%/36.2%	0.02
Protocol: Agonist	3.8%/2.5%/8.9%	0.28	4.0%/3.8%/4.1%	0.02
Trigger: GnRH Agonist	82.2%/85.9%/64.9%	0.50	79.9%/80.4%/81.1%	0.01
Trigger: hCG (including dual trigger)	17.8%/14.0%/35.0%	0.50	20.1%/19.6%/18.9%	0.01

*Note:* Data are presented as mean values (standard deviation: SD) for continuous variables and numbers (percentages) for categorical variables. Covariate balance between the three groups (Follitropin alpha, Follitropin delta, and Urinary hMG) was evaluated using the maximum standardized mean difference (Max SMD) among pairwise comparisons; an absolute Max SMD value below 0.10 was deemed to reflect sufficient balance. Additionally, the distributions of ovarian stimulation protocols and trigger methods are reported. The C‐statistics for the propensity score models used for matching were 0.62 (Follitropin alpha vs. Delta), 0.73 (Follitropin delta vs. hMG), and 0.64 (Follitropin alpha vs. hMG), indicating adequate model fit without violating the positivity assumption.

Abbreviations: AFC: antral follicle count; AMH: anti‐Müllerian hormone; ART: assisted reproductive technology; GnRH: gonadotropin‐releasing hormone; hMG: human menopausal gonadotropin; PPOS: progestin‐primed ovarian stimulation.

As shown in Table 2, although the duration of stimulation differed statistically among the groups, the absolute differences were small (ranging from 8.1 to 8.5 days) and clinically negligible (Cohen's *d* ≤ 0.17). The total gonadotropin dose was significantly lower in the follitropin alpha group (1870 ± 1070 IU) than in the hMG group (2310 ± 1340 IU; *p* < 0.01, *d* = 0.36).

**TABLE 2:** Comparative outcomes of ovarian stimulation across treatment groups.

Outcome	Follitropin alpha (*n* = 2549)	Follitropin alpha vs. Follitropin delta	Follitropin delta (*n* = 2549)	Follitropin delta vs. hMG	Urinary hMG (*n* = 2549)	Follitropin alpha vs. hMG
Duration of stimulation (days)	8.3 ± 3.3	0.07 (0.05)	8.1 ± 1.9	< 0.01 (0.17)	8.5 ± 2.2	< 0.01 (0.08)
Total Dose (μg, IU)	1870 ± 1070	N.A	76.2 ± 23.5	N.A	2310 ± 1340	< 0.01 (0.36)
FSH after stimulation (mIU/mL)	15.4 ± 6.2	0.45 (0.02)	15.6 ± 6.9	< 0.01 (0.55)	18.8 ± 5.2	< 0.01 (0.61)
Ovarian enlargement (All patients) (%)	37.4%	1.06 (0.96–1.17)	35.3%	0.78 (0.72–0.86)	45.0%	0.83 (0.76–0.91)
Ovarian enlargement (HCG trigger) (%)^a^	20.1%	1.24 (0.88–1.75)	16.2%	0.54 (0.39–0.74)	30.2%	0.67 (0.50–0.89)

*Note:* Results of continuous variables are reported as the mean ± SD and *p*‐value with Cohen's *d*. Results of categorical variables are written in percentage and risk ratio with a 99% confidence interval. Due to differences in measurement units for follitropin delta, dose comparisons were performed exclusively between follitropin alpha and hMG. Ovarian enlargement was defined by a simplified classification as a maximum ovarian diameter ≥ 5 cm at the time of oocyte retrieval. Results were considered statistically significant if the *p*‐value was < 0.01, or if the 99% confidence interval did not include 1.0. Cohen's *d* indicates that a value of 0.2–0.5 indicates a small difference, 0.5–0.8 suggests a moderate difference, and 0.8 or above represents a large difference.

^a^ The analysis of ovarian enlargement (HCG trigger) was restricted to patients who received an hCG trigger (including dual trigger), with the following sample sizes: Follitropin alpha (*n* = 512), Follitropin delta (*n* = 499), and Urinary hMG (*n* = 483).

Post‐stimulation serum FSH levels were significantly higher in the hMG group (18.8 ± 5.2 mIU/mL) compared with both the follitropin alpha group (15.4 ± 6.2 mIU/mL; *p* < 0.01, *d* = 0.61) and the follitropin delta group (15.6 ± 6.9 mIU/mL; *p* < 0.01, *d* = 0.55).

The incidence of ovarian enlargement ≥ 5 cm at the time of oocyte retrieval was 37.4% in the follitropin alpha group, 35.3% in the follitropin delta group, and 45.0% in the hMG group. A lower observed incidence was noted in both recombinant FSH groups compared with the hMG group (alpha vs. hMG: RR 0.83, 99% CI 0.76–0.91; delta vs. hMG: RR 0.78, 99% CI 0.72–0.86). A similar directional pattern was observed in the subgroup restricted to hCG‐triggered cycles (Table 2), though this remains a descriptive observation only. However, because clinically diagnosed OHSS was not evaluated, this surrogate measure alone does not establish a clinical safety benefit.

Table 3 summarizes the outcomes on oocyte retrieval and subsequent embryological outcomes. Regarding the primary endpoint (number of oocytes retrieved), the overall results were statistically indeterminate across analytical approaches. While the PSM analysis observed a significantly higher number of oocytes in the follitropin alpha group (8.8 ± 6.7) compared with follitropin delta (8.0 ± 5.5; *p* < 0.01, *q* < 0.01) and the hMG group (8.3 ± 5.9; *p* < 0.01, *q* < 0.01), this finding was not robust, as the effect sizes were very small (Cohen's *d* = 0.13 and 0.07, respectively) and the difference was not confirmed in the IPW analysis. Regarding embryological outcomes, both recombinant preparations yielded significantly higher numbers of blastocysts (alpha: 3.3 ± 3.2; delta: 3.1 ± 2.9) compared to the urinary hMG group (2.7 ± 2.6; *p* < 0.01, *q* < 0.01), while no significant difference was observed between the two recombinant groups. Similarly, the number of high‐quality blastocysts was significantly higher in both follitropin alpha (1.2 ± 1.6) and follitropin delta (1.2 ± 1.5) groups than in the hMG group (0.8 ± 1.3; *p* < 0.01, *q* < 0.01).

**TABLE 3:** Values are presented as mean ± standard deviation (SD) per cycle for continuous variables, or as percentages for categorical variables.

Outcome	Follitropin alpha (*n* = 2549)	Follitropin alpha vs. Follitropin delta	Follitropin delta (*n* = 2549)	Follitropin delta vs. hMG	Urinary hMG (*n* = 2549)	Follitropin alpha vs. hMG	IPW: Alpha vs. Delta (IRR, 99% CI, *p*)	IPW: hMG vs. Delta (IRR, 99% CI, *p*)
Number of oocytes retrieved	8.8 ± 6.7	< 0.01, < 0.01 (0.13)	8.0 ± 5.5	0.04, 0.05 (0.06)	8.3 ± 5.9	< 0.01, < 0.01 (0.07)	1.05 (0.98–1.12), *p* = 0.07	1.03 (0.96–1.11), *p* = 0.24
Number of fertilized oocytes	5.8 ± 4.6	< 0.01, < 0.01 (0.14)	5.2 ± 4.0	< 0.01, 0.01 (0.07)	5.5 ± 4.2	0.01, 0.02 (0.07)	—	—
Number of cleavage stage embryos	5.3 ± 4.3	< 0.01, < 0.01 (0.13)	4.7 ± 3.7	0.06, 0.06 (0.05)	4.9 ± 3.8	< 0.01, < 0.01 (0.08)	—	—
Number of embryos cultured to blastocyst stage	5.5 ± 4.2	< 0.01, < 0.01 (0.15)	4.9 ± 3.6	0.10, 0.10 (0.05)	4.7 ± 3.5	< 0.01, < 0.01 (0.20)	—	—
Number of blastocysts	3.3 ± 3.2	0.04, 0.05 (0.06)	3.1 ± 2.9	< 0.01, < 0.01 (0.16)	2.7 ± 2.6	< 0.01, < 0.01 (0.21)	1.00 (0.93–1.07), *p* = 0.95	0.86 (0.80–0.94), *p* < 0.01
Number of high‐quality blastocysts	1.2 ± 1.6	0.31, 0.31 (0.03)	1.2 ± 1.5	< 0.01, < 0.01 (0.26)	0.8 ± 1.3	< 0.01, < 0.01 (0.23)	—	—
Blastocysts formation rate (%)	64.1	0.95 (0.92–0.97)	67.8	1.13 (1.10–1.15)	60.2	1.06 (1.04–1.09)	—	—
High‐quality blastocysts formation rate (%)	22.6	0.86 (0.81–0.91)	26.2	1.40 (1.31–1.49)	18.8	1.20 (1.13–1.29)	—	—

*Note:* For the propensity score‐matched (PSM) cohort, Welch's *t*‐test was used to compare the means of continuous variables, and the *χ* ^2^ test was used for categorical variables. Both raw *p*‐values and False Discovery Rate (FDR) adjusted *q*‐values were calculated to assess statistical significance. Continuous outcomes are shown as mean ± SD with *p*‐value, *q*‐value (Cohen's *d*), while categorical outcomes are expressed as percentages with risk ratios (99% confidence intervals). Cohen's *d* values of 0.2–0.5 indicate a small effect, 0.5–0.8 a moderate effect, and ≥ 0.8 a large effect. Results were considered statistically significant if both the *p*‐value and *q*‐value were < 0.01, or if the 99% confidence interval did not include 1.0. Additionally, to provide transparent comparison across analytical models, the results of the inverse probability weighting (IPW) sensitivity analysis for the primary outcome (oocyte yield) and key secondary outcome (blastocyst yield) are presented in the rightmost columns as incidence rate ratios (IRR) with 99% confidence intervals and *p*‐values.

Table S1 presents the outcomes of cluster‐robust regression analyses, which were performed to confirm the consistency of the main findings reported in Tables 2 and 3. These models accounted for repeated cycles at the patient level and confirmed the robustness of the results. Follitropin alpha was associated with a significantly higher oocyte yield compared with follitropin delta (IRR 1.10, 99% CI 1.02–1.18). In contrast, urinary hMG was associated with significantly fewer blastocysts (IRR 0.86, 99% CI 0.77–0.95), fewer high‐quality blastocysts (IRR 0.69, 99% CI 0.59–0.80), and a higher incidence of ovarian enlargement ≥ 5 cm (OR 1.50, 99% CI 1.20–1.87). Consistent with these findings, differences between follitropin alpha and delta in blastocyst‐related outcomes were not statistically significant after accounting for patient‐level clustering, indicating that the cycle‐level associations were not robust to patient‐level adjustment.

As shown in the rightmost columns of Table 3, the inverse probability weighting (IPW) sensitivity analysis on the full unmatched cohort (*n* = 10 997) was inconsistent with the PSM analysis regarding the primary outcome: the difference in oocyte yield between follitropin alpha and delta was not statistically significant in the weighted model (IRR 1.05, 99% CI 0.98–1.12; *p* = 0.07), further supporting the indeterminate nature of this finding. For secondary outcomes, the IPW estimates were broadly consistent with the PSM findings, with the lower blastocyst yield in the hMG group remaining statistically significant (IRR 0.86, *p* < 0.01).

Table 4 summarizes clinical outcomes following frozen–thawed single blastocyst transfer. Clinical pregnancy rates were similar across groups (45.9% for follitropin alpha, 43.1% for follitropin delta, and 46.8% for hMG), with no significant differences (alpha vs. delta: RR 1.07, 99% CI 0.97–1.17; delta vs. hMG: RR 0.92, 99% CI 0.84–1.01; alpha vs. hMG: RR 0.98, 99% CI 0.90–1.07). Spontaneous abortion and ongoing pregnancy/live birth rates were also comparable among the groups.

**TABLE 4:** Comparative analysis of clinical outcomes following frozen–thawed single blastocyst transfer among three groups.

Outcome	Follitropin alpha	Follitropin alpha vs. Follitropin delta	Follitropin delta	Follitropin delta vs. hMG	Urinary hMG	Follitropin alpha vs. hMG
Cycles	2113		2003		2105	
Clinical pregnancy (%)	970 (45.9)	1.07 (0.97–1.17)	863 (43.1)	0.92 (0.84–1.01)	985 (46.8)	0.98 (0.90–1.07)
Spontaneous Abortion (%)	247 (25.5)	1.16 (0.94–1.45)	189 (21.9)	0.89 (0.72–1.11)	242 (24.6)	1.04 (0.85–1.27)
Ongoing pregnancy or live birth (%)	723 (34.2)	1.02 (0.91–1.14)	674 (33.6)	0.95 (0.85–1.07)	743 (35.3)	0.97 (0.87–1.08)

*Note:* Values are presented as numbers accompanied by percentages, while comparisons are expressed as risk ratios with corresponding 99% confidence intervals.

## 4 Discussion

In this comprehensive retrospective study, the primary outcome regarding oocyte yield was statistically indeterminate across analytical approaches. Although the initial propensity score‐matched (PSM) analysis observed a statistically higher number of oocytes in the follitropin alpha group compared with follitropin delta and hMG, the effect sizes were very small. More importantly, this observation was not robust, as a sensitivity analysis using inverse probability weighting (IPW) did not confirm a statistically significant difference in oocyte yield between follitropin alpha and delta. Therefore, these findings suggest broadly comparable clinical efficacy among the three gonadotropin preparations, without a clear superiority in oocyte yield. Exploratory analyses of secondary outcomes indicated that both recombinant preparations were associated with a lower incidence of ovarian enlargement ≥ 5 cm at the time of oocyte retrieval compared with hMG. Similar patterns of ovarian response have been reported in previous randomized controlled trials and observational studies [13, 14]. In addition, subgroup analysis restricted to cycles using an hCG trigger demonstrated that this difference persisted in a higher‐risk population. However, because clinically diagnosed OHSS was not evaluated in this study, ovarian enlargement ≥ 5 cm represents strictly an anatomical surrogate. Therefore, we explicitly emphasize that these findings do not establish a clinical safety benefit or a validated clinical safety advantage.

In this study, serum FSH levels in both the follitropin alpha and follitropin delta groups were lower than those observed in the hMG group. Additionally, the total dose of follitropin alpha administered was significantly lower than hMG (1870 ± 1070 IU vs. 2310 ± 1340 IU, *p* < 0.01).

Given that the number of oocytes retrieved did not differ significantly among the groups, these findings suggest that r‐FSH preparations were observed to be associated with comparable ovarian stimulation outcomes to hMG, despite lower observed serum FSH levels and total gonadotropin doses. However, it is crucial to emphasize that post‐stimulation serum FSH levels are not directly comparable between rFSH and hMG due to inherent differences in LH activity and pharmacodynamics. Therefore, any hypothesized relationship to clinical safety outcomes remains speculative and cannot be substantiated by the present data.

In the present study, both recombinant FSH preparations were observationally associated with a higher mean number of blastocysts per cycle compared with hMG across multiple analytical approaches. These findings are consistent with several previous reports suggesting differences in embryological outcomes between recombinant and urinary gonadotropin preparations [15, 16, 17, 18]. However, other studies have reported no significant differences [19, 20], and in certain clinical contexts such as low serum LH concentration, additional LH activity may be beneficial [21]. In the present study, differences between follitropin alpha and delta in blastocyst‐related outcomes were not retained after accounting for patient‐level clustering. Similarly, Nyboe Andersen et al. reported no difference between follitropin delta and follitropin beta in the total number of blastocysts or good‐quality blastocysts [6]. Therefore, whether the pharmacokinetic characteristics exert any direct effect on embryo morphological or developmental quality remains unclear, as conclusive clinical evidence is lacking [22].

This study found no significant differences among the three groups in clinical pregnancy rate, ongoing pregnancy, or live birth rate after frozen–thawed single blastocyst transfer. Consistent with these findings, earlier reports indicated no notable difference in clinical pregnancy rates in either fresh embryo transfer cycles [6, 7, 8] or frozen–thawed embryo transfer cycles [23] following follitropin delta administration. Conversely, Bühler et al. reported higher cumulative pregnancy and live birth rates in the follitropin alpha group compared to the hMG group [4]. Moreover, recent multi‐center PSM analyses have indicated that the follitropin delta group achieved higher cumulative pregnancy rates than the follitropin alpha group [24], suggesting that pregnancy outcomes may vary across studies. Such discrepancies among reports may be attributed to the multifactorial nature of pregnancy and childbirth, which are influenced not only by the quality of the fertilized embryo but also by uterine factors and other physiological conditions. Therefore, when aiming to evaluate the embryo itself in isolation, it would be desirable to incorporate chromosomal assessment in addition to conventional morphological grading. However, it is important to note that, to date, no studies have specifically evaluated preimplantation genetic testing (PGT) outcomes as a primary endpoint in relation to gonadotropin preparations. Therefore, further research in this area is warranted.

This study has several limitations. First, its retrospective design inherently carries a risk of selection bias. Although PSM was applied to adjust for background characteristics as much as possible, residual confounding due to unmeasured variables cannot be completely excluded. To further assess the robustness of our findings, we additionally performed inverse probability weighting (IPW) as a sensitivity analysis, which yielded results that were inconsistent with the PSM findings regarding the primary outcome, further supporting the indeterminate nature of the oocyte yield results.

Second, a potential secular trend bias exists because follitropin delta was introduced only after 2021. To minimize this bias, we restricted our analysis to the period from 2021 to 2024, ensuring that all three treatment options were available throughout the study duration. Additionally, we applied PSM adjusting for relevant covariates including body weight and trigger method, as well as regression models with cluster‐robust standard errors.

Third, the choice of the number of retrieved oocytes as the primary endpoint represents a limitation. While this outcome reflects ovarian response and is directly related to the efficacy of FSH preparations, it remains an intermediate outcome. Clinically most relevant endpoints are ongoing pregnancy and live birth. However, these outcomes are strongly influenced by maternal and uterine factors, and the use of preimplantation genetic testing (PGT) to isolate embryo‐related effects is rare in Japan. For this reason, live birth could not be feasibly adopted as the primary endpoint in this study. Nevertheless, our conclusions should be interpreted with caution and not be overextended as evidence of superiority in final reproductive outcomes.

Fourth, excessive ovarian response was evaluated using an anatomical surrogate definition based on ovarian diameter (≥ 5 cm) at the time of oocyte retrieval. Importantly, we did not evaluate the incidence of clinical OHSS in this study, and this surrogate does not necessarily capture the full clinical spectrum of the syndrome.

Fifth, while multiple embryological outcomes were analyzed, only exploratory outcomes were subjected to multiplicity adjustment using the false discovery rate (FDR). The primary and prespecified secondary outcomes were reported with raw *p*‐values. Although this approach follows standard practice, the residual risk of type I error cannot be fully excluded.

Finally, this study was conducted at a single institution, with 99% of participants being Asian, predominantly Japanese. Consequently, the generalizability of the findings to patient populations of other racial or ethnic backgrounds may be limited.

## 5 Conclusions

In this large retrospective analysis, the primary outcome regarding oocyte yield was statistically indeterminate across different analytical approaches, and the observed differences were clinically marginal. Exploratory analyses of secondary outcomes indicate that both recombinant preparations (follitropin delta and alpha) were observationally associated with a greater number of blastocysts and a lower incidence of ovarian enlargement ≥ 5 cm at oocyte retrieval compared with hMG. These hypothesis‐generating findings suggest potential pharmacological differences among gonadotropin preparations. However, because the lower incidence of ovarian enlargement ≥ 5 cm represents only an anatomical surrogate and clinically diagnosed OHSS was not evaluated, these findings do not establish clinical superiority or a clinical safety benefit. Future prospective studies are essential to confirm these observations.

## Ethics Statement

All procedures in this study were in accordance with the ethical standards of the Ethical Committee in accordance with the ethical principles that have their origin in the Declaration of Helsinki 1964 and its later amendments. This study was approved by the Ethical Committee of Hanabusa Women's Clinic, which consists of members chosen by our institute and a third‐party medical institute (approval number: 2025‐06).

## Consent

All patients were well informed and written informed consent was obtained prior to the treatment period.

## Conflicts of Interest

The authors declare no conflicts of interest.

## Supporting information


**Figure S1:** Propensity score distributions before and after matching. The histograms and kernel density plots show the distribution of propensity scores for each pairwise comparison. (Top) Follitropin alpha vs. Follitropin delta before matching (left) and after matching (right). (Bottom) Urinary hMG vs. Follitropin delta before matching (left) and after matching (right). The overlapping distributions in the post‐matching plots demonstrate that the matching process successfully balanced the covariate profiles between the treatment groups, ensuring sufficient common support.

**Table S1:** Cluster‐robust regression analysis of oocyte yield, blastocyst development, high‐quality blastocyst formation, and ovarian enlargement ≥ 5 cm at oocyte retrieval among follitropin alpha, follitropin delta, and urinary hMG. Regression models were fitted with cluster‐robust standard errors at the patient level to account for repeated cycles. Analyses were conducted after propensity score matching (PSM). Incidence rate ratios (IRR) were estimated using negative binomial regression for count outcomes (oocytes, blastocysts, high‐quality blastocysts), and odds ratios (OR) were estimated using logistic regression for the binary outcome (ovarian enlargement). The follitropin delta group was used as the reference. Statistically significant results were defined as *p* < 0.01 or 99% CI not including 1.0.

## Data Availability

The data that support the findings of this study are available from the corresponding author upon reasonable request.
