# Supplementary figures and images for: Comparative Effectiveness of Follitropin Delta, Follitropin Alpha, and hMG in ART Cycles: A Single‐Center Retrospective Cohort Study With Propensity Score Matching
Source: Reprod Med Biol. 2026 May 13;25(1):e70060. doi: 10.1002/rmb2.70060 (PMC13172277; doi:10.1002/rmb2.70060)

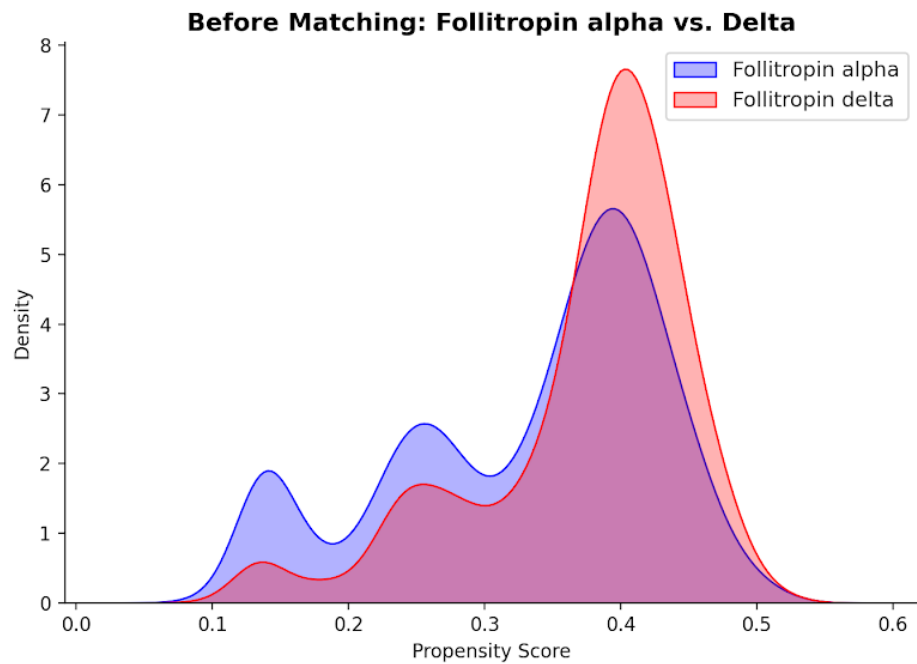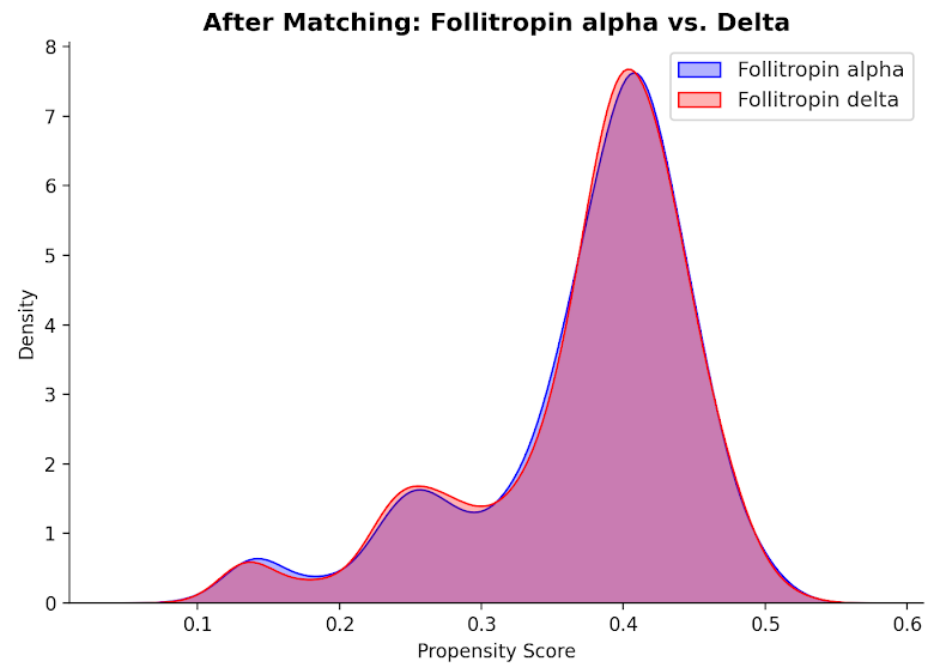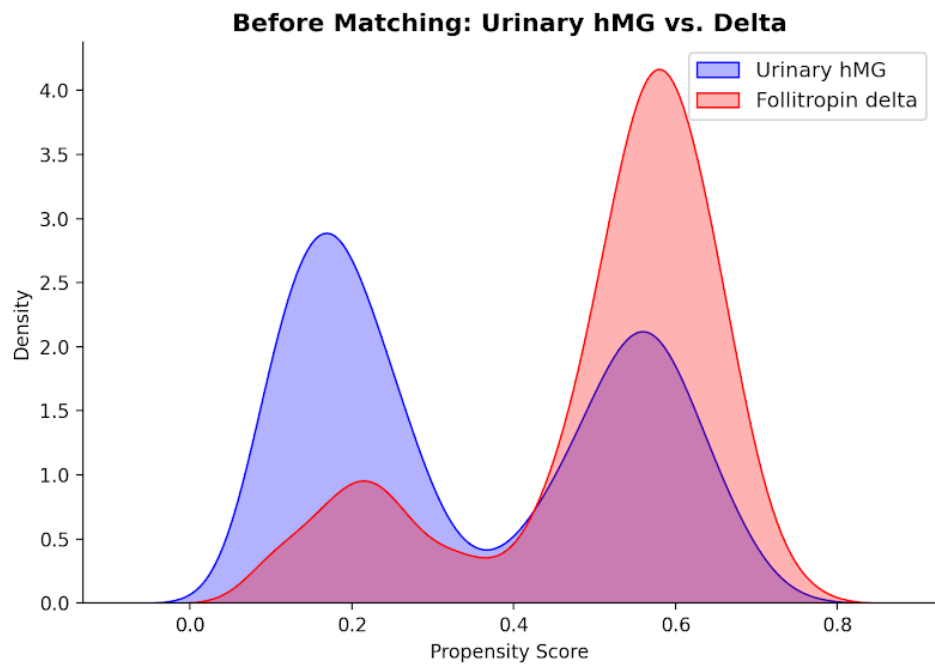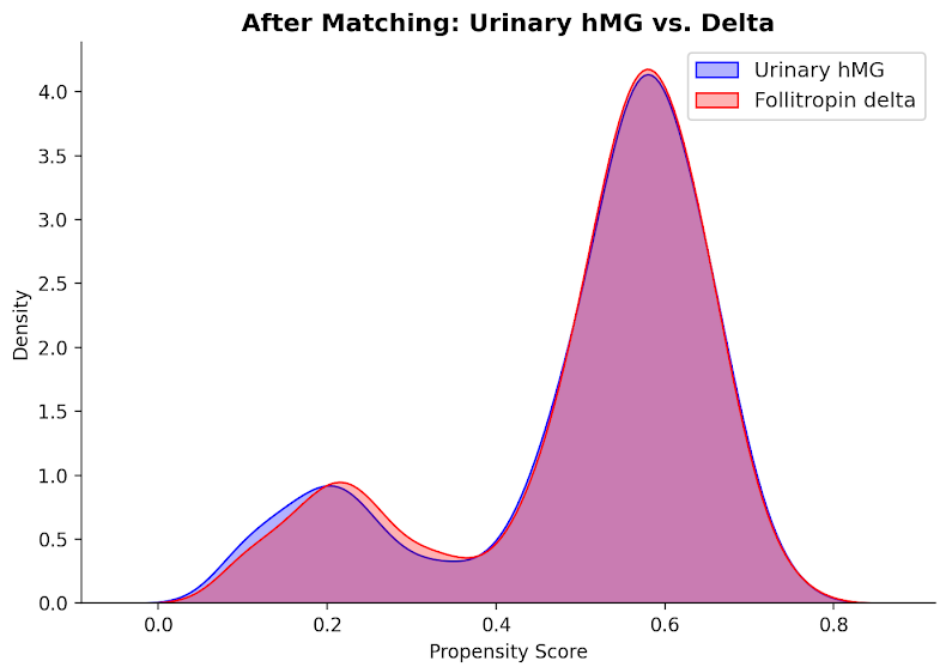

Supplement: Supplementary file 1 — Figure S1: Propensity score distributions before and after matching. The histograms and kernel density plots show the distribution of propensity scores for each pairwise comparison. (Top) Follitropin alpha vs. Follitropin delta before matching (left) and after matching (right). (Bottom) Urinary hMG vs. Follitropin delta before matching (left) and after matching (right). The overlapping distributions in the post‐matching plots demonstrate that the matching process successfully balanced the covariate profiles between the treatment groups, ensuring sufficient common support. [file RMB2-25-e70060-s002.pdf]
