# Supplementary material for: Comparative Effectiveness of Follitropin Delta, Follitropin Alpha, and hMG in ART Cycles: A Single‐Center Retrospective Cohort Study With Propensity Score Matching
Source: Reprod Med Biol. 2026 May 13;25(1):e70060. doi: 10.1002/rmb2.70060 (PMC13172277; doi:10.1002/rmb2.70060)
Supplement: Supplementary file 2 — Table S1: Cluster‐robust regression analysis of oocyte yield, blastocyst development, high‐quality blastocyst formation, and ovarian enlargement ≥ 5 cm at oocyte retrieval among follitropin alpha, follitropin delta, and urinary hMG. Regression models were fitted with cluster‐robust standard errors at the patient level to account for repeated cycles. Analyses were conducted after propensity score matching (PSM). Incidence rate ratios (IRR) were estimated using negative binomial regression for count outcomes (oocytes, blastocysts, high‐quality blastocysts), and odds ratios (OR) were estimated using logistic regression for the binary outcome (ovarian enlargement). The follitropin delta group was used as the reference. Statistically significant results were defined as p < 0.01 or 99% CI not including 1.0. [file RMB2-25-e70060-s001.docx]

Supplementary Table S1. Cluster-robust regression analysis of oocyte yield, blastocyst development, high-quality blastocyst formation, and ovarian enlargement ≥5 cm at oocyte retrieval among follitropin alpha, follitropin delta, and urinary hMG. Regression models were fitted with cluster-robust standard errors at the patient level to account for repeated cycles. Analyses were conducted after propensity score matching (PSM). Incidence rate ratios (IRR) were estimated using negative binomial regression for count outcomes (oocytes, blastocysts, high-quality blastocysts), and odds ratios (OR) were estimated using logistic regression for the binary outcome (ovarian enlargement). The follitropin delta group was used as the reference. Statistically significant results were defined as p < 0.01 or 99% CI not including 1.0.

| Outcome | Comparison (vs delta) | Effect size (IRR/OR) | 99% CI | p-value |
| --- | --- | --- | --- | --- |
| Number of Oocytes | Alpha | 1.10 | 1.02 – 1.18 | <0.01 |
|  | hMG | 1.04 | 0.96 – 1.12 | 0.18 |
| Number of blastocysts | Alpha | 1.06 | 0.97 – 1.16 | 0.12 |
|  | hMG | 0.86 | 0.77 – 0.95 | <0.01 |
| Number of high-quality blastocysts | Alpha | 0.96 | 0.85 – 1.09 | 0.44 |
|  | hMG | 0.69 | 0.59 – 0.80 | <0.01 |
| Ovarian enlargement | Alpha | 1.09 | 0.90 – 1.33 | 0.23 |
|  | hMG | 1.50 | 1.20 – 1.87 | <0.01 |
